# Built Environment and Its Influences on Walking among Older Women: Use of Standardized Geographic Units to Define Urban Forms

**DOI:** 10.1155/2012/203141

**Published:** 2012-08-22

**Authors:** Vivian W. Siu, William E. Lambert, Rongwei Fu, Teresa A. Hillier, Mark Bosworth, Yvonne L. Michael

**Affiliations:** ^1^Population Research Center and Institute of Metropolitan Studies, Portland State University, P.O. Box 751, Portland, OR 97207, USA; ^2^Department of Public Health and Preventive Medicine, Oregon Health & Science University, 3181 SW Sam Jackson Park Road, Mail Code CB 669, Portland, OR 97239, USA; ^3^Kaiser Permanente Center for Health Research, Northwest/Hawaii, 3800 N. Interstate Avenue, Portland, OR 97227, USA; ^4^Metro Data Resource Center, 600 NE Grand Avenue, Portland, OR 97232, USA; ^5^Department of Epidemiology and Biostatistics, Drexel University School of Public Health, 1505 Race Street, MS 1033, Philadelphia, PA 19102, USA

## Abstract

Consensus is lacking on specific and policy-relevant measures of neighborhood attributes that may affect health outcomes. To address this limitation, we created small standardized geographic units measuring the transit, commercial, and park area access, intersection, and population density for the Portland, Oregon metropolitan area. Cluster analysis was used to identify six unique urban forms: central city, city periphery, suburb, urban fringe with poor commercial access, urban fringe with pool park access, and satellite city. The urban form information was linkable to the detailed physical activity, health, and socio-demographic data of 2,005 older women without the use of administrative boundaries. Evaluation of the relationship between urban forms and walking behavior indicates that older women residing in city center were more likely to walk than those living in city periphery, suburb communities, and urban fringe with poor commercial access; however, these women were not significantly more likely to walk compared to those residing in urban fringe with poor park access or satellite city. Utility of small standardized geographic units and clusters to measure and define built environment support research investigating the impact of built environment and health. The findings may inform environmental/policy interventions that shape communities and promote active living.

## 1. Introduction

Urban forms, or areas with different combinations of built environment characteristics, are believed to influence people's daily physical activities. Urban sprawl correlates with increasing automobile dependency and sedentary lifestyle over the past half century [1]. In response, planning efforts to promote New Urbanism and Smart Growth have grown in recent years [2]. At the same time, the number and proportion of older adults in the United States are also increasing [3]. Older adults represent a population that could benefit from these planning efforts; in general, older adults engage in relatively low levels of physical activity [4, 5] and in low intensity activities such as walking and gardening for exercise [6], compared to younger adults. Older women are of particular concern, since women tend to have lower levels of physical activity than men in any age group [4, 7]. Women also tend to live longer than men, so they constitute a large share of the vulnerable population. Positive associations were found between accessibility of facilities, housing density, population density, and the level of walking in several studies that focused on older populations [8–12], and particularly among older women [13, 14] in different neighborhoods and cities across the United States. 

Challenges with quantifying built environment measurements, including inconsistent attribute definitions, different scales of data, and varying data quality by jurisdictions, make it difficult to compare between studies designed to quantify the benefits of certain urban form in relation to physical activity. Studies with built environments measured at the individual level typically gathered information by surveying individuals' perceptions of the environment [11, 12, 15, 16], by aggregating neighborhood measures from secondary data such as Census [10, 13, 15–21], or by measuring these characteristics within a certain distance of the subjects' residences [11, 13, 16, 22–25]. Few previous studies have quantified the built environment attributes objectively at high resolution or used cluster analysis to identify different urban forms [17, 26–28]. 

These research gaps provided an opportunity for the current study to expand on the existing literature investigating the association between built environments and walking among older women by developing refined built environment measures in order to identify distinct urban forms. A better understanding of the relationship between built environments and walking behaviors could lead to urban design and planning policies that promote walking, thereby generating public health benefits. 

We hypothesized that a compact urban form promotes walking among older women. Compact neighborhoods with high population density, high street connectivity, and close proximity to transit services, commercial areas, and parks were posited to be favorable to pedestrian activity. High connectivity and convenient access to amenities were hypothesized to shape transportation choices and encourage utilitarian and leisure walking, and high population density was anticipated to support investment of infrastructure and amenities. Older women residing in these compact areas are expected to walk more than those residing in remote areas with low population density, poor street connectivity, and poor accessibility to transit services, commercial areas, and park areas. 

## 2. Materials and Methods

Our analysis used data from participants in the Portland, Oregon, site of the Study of Osteoporotic Fractures (SOF), a national multicenter observational study of healthy, community-dwelling women age 65 years and older recruited in 1986 from four metropolitan areas in the United States. Women were recruited irrespective of bone mineral density and fracture history; those unable to walk without assistance and those with bilateral hip replacements were excluded. All women provided written consent, and the SOF study was approved by each site's institutional review board. Although only baseline data were examined for the current study, prospective data collection included clinical exams and questionnaires to assess longitudinal change in anthropometric and demographic factors, medical history, and information on functioning, quality of life, and lifestyle [29]. Measurement and quality control procedures for the SOF cohort are detailed elsewhere [30]. 

The subset of SOF participants from Portland, Oregon, totaled 2,419 subjects. In the current analysis, we excluded 347 women (14.3%) who resided outside of the urban growth boundary (UGB) and 67 women (2.8%) whose residence could not be geocoded (e.g., P.O. Box given for address). Thus, the analytic sample was restricted to 2,005 subjects who resided within the Portland UGB, representing 82.9% of the total Portland, Oregon, SOF cohort.

### 2.1. Built Environment Measures

Six neighborhood measures were created using the existing Portland Metro's Regional Land Information System (RLIS) administrative data and ArcGIS 9.3 (ESRI, Redlands, CA, USA). Among this set of six measures, two measures of accessibility to transit services were included: distance to bus stop and distance to light rail station. Two measures of residential/commercial/recreational land use mix were included: distance to commercial area and distance to park area. One measure of street connectivity was created: street intersection density. One measure of residential distribution was created: population density. Spatial information was unavailable for 1986; therefore data from the earliest available year were used. The 1988 archival transit data from TriMet, the local transit agency, was digitized to develop the distance to bus stop and light rail station variables. Metro-maintained zoning and park data from 1990 were used to create the distance to commercial area and park area variables. A 1988 streets file was established for measuring the intersection density variable, and 1990 Census block population data was adopted for developing the population density variable. 

The six built environment variables were developed in a raster environment for the entire Portland metro area. Each grid cell was set at 264 feet^2^ (or 80 meters^2^), the length for each side of the grid would take roughly one minute of brisk walking to complete. Usage of high-resolution grid cells to quantify built environment attributes minimized the geographic unit of measure. The refined geographic units for establishing built environment measures lessened the potential bias associated with the use of coarsely grouped measures, in which neighborhood characteristics might be wrongly assumed as the experience for the individuals (i.e., the ecological fallacy). The high-resolution cell size also facilitated linkage of localized resident neighborhood measures to the subjects based on their mapped residential address location. 

The neighborhood measures were standardized to ensure comparability among measures with different scales. The four distance variables (accessibility to the closest bus stop, light rail station, commercial area, and park area) were measured in Euclidean distance; shorter distance indicated better accessibility. The two density variables, intersection density and population density, were created using the kernel density function, accounting for the number of street intersections within a quarter mile buffer from each grid cell and the number of persons in a one mile buffer; higher number indicated greater density.

The reliability and validity of the built environment measures were evaluated using a combination of GIS analysis processes and statistical tests. Vector/direct distance and density measurements for the six attributes were established for the study sample and compared to the raster approach. Various tests and graphs used for assessing the level of agreement between built environment measures developed through the raster method, and the vector method indicated reasonable agreement [31], meaning that the distance and density measurements created using the raster and the more standard and commonly adopted vector approaches were similar.

### 2.2. Walking

SOF participants reported the total number of blocks walked daily (12 blocks = 1 mile) for utilitarian and for leisure purposes; we added these measures to create the overall number of blocks walked daily. Since a fair share of the participants (11%) reported no walking at all, this variable was dichotomized into walking less than five blocks a day, and walking five or more blocks a day. Five blocks is equivalent to about 400 meters or a quarter of a mile. This amount of walking has been found to provide health benefit to sedentary older adults [32]. As a result of this classification, 65% of our subjects (*n* = 1,572) walked five or more blocks a day.

### 2.3. Statistical Analysis

We used k-median statistical clustering to group grid cells with similar values of accessibility to transit, land use mix, street connectivity, and residential density into clusters representing types of urban-form. The Calinski-Harabasz stopping rule was used to determine the optimal number of clusters that would maximize the between group differences while minimizing the within group differences of the built environment measures considered [33]. Grid cells with similar composition of neighborhood attributes were aggregated into the same group, converting the six continuous built environment measures into six categories of exposure corresponding to distinct urban forms.

Subject characteristics were summarized using descriptive statistics. Multivariable logistic regression models were used to assess the association between urban-form and walking while controlling for confounding variables. Potential confounding variables included individual characteristics of the women: age, years of education, marital status, overall exercise (Kcal/week), BMI (kg/m^2^), smoking (pack years), self-reported health, history of stroke, and history of arthritis/rheumatism. Additional potential confounding variables included block group level neighborhood characteristics from the Census: demographic composition, education, occupation, poverty, income, and household type. All potential confounders with a univariate *P* value less than 0.25 were considered in the preliminary model. Variables were removed from the model one by one beginning with the variable with the highest *P* value until all variables in the model were significant at *α* = 0.05. The pairwise comparisons of the impact on walking by different urban forms were also explored to identify urban forms that might have impacted walking differently. All statistical analyses were conducted using Stata 11 (StataCorp, College Station, TX, USA).

## 3. Results

The k-median cluster analysis resulted in six distinct urban-form clusters across the metro region. The medians and interquartile ranges of built environment measures for each cluster are summarized in Table 1. Clusters 1, 2, and 3 showed a gradual decrease in accessibility and density for all built environment attributes: with Cluster 1 being the “best” urban-form with close proximity to transit, commercial areas, and park areas. Intersection and population density in Cluster 1 were also the highest of the six clusters. The accessibility and density decreased from the central core to Cluster 2 and dropped even further for Cluster 3. Although Clusters 4, 5, and 6 generally had poorer accessibility to amenities and lower density than Cluster 3, there were certain attributes in each of these clusters that were similar to another cluster and that provide some insight as to how specific attributes influence walking habits. For instance, Clusters 4 and 5 shared low intersection and population density, and they had similar measures for access to bus or light rail services; they differed in that Cluster 4 had the greatest distance to commercial areas while Cluster 5 had the greatest distance to park areas. Cluster 6 was similar to Cluster 3 in terms of access to commercial areas and density, but it had the greatest distance to transit services among the six clusters.

**Figure 1:**
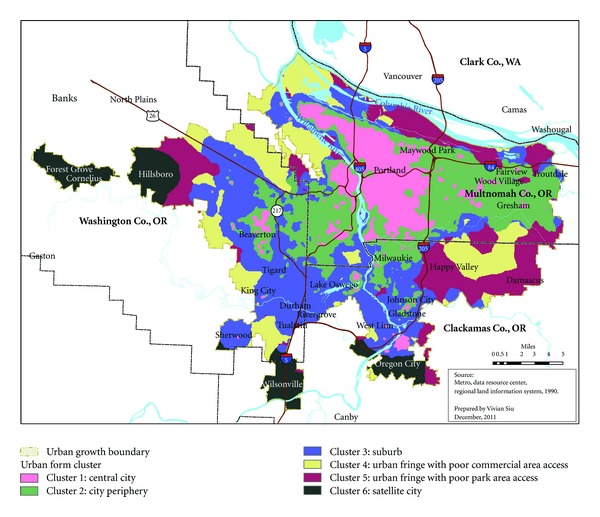
Spatial representation of urban-form clusters, Portland metro area.

 The resulting clusters were mapped into ArcGIS to evaluate face validity (Figure 1). The map demonstrates reasonable clustering with grid cells assigned to the same group being spatially continuous; the distribution of the six clusters was also sensible and consistent with our knowledge of the environmental characteristics of the metro region.

The walking behavior of older women varied by purpose of walking and type of cluster (Table 2). Generally, older women who walked for leisure purposes tend to walk more than those who walked only for utilitarian purpose in each of the six clusters. The relative variation in walking by urban-form typology demonstrated a similar pattern for those who walked for utilitarian purposes, for leisure purposes, as well as for both purposes combined. Specifically, the central city consistently ranked one of the highest while the urban fringe with poor commercial area access ranked the lowest among the six clusters in median number of daily blocks walked, regardless of walking purposes.

Table 3 provides results from the multivariable logistic regression estimating the association between urban-form and walking. Compared to women who lived in the central city, those who lived in the urban fringe with poor commercial area access, city periphery, and suburbs were significantly less likely to walk each day (adjusted odds ratios (ORs) ranged from 0.40 for urban fringe to 0.69 for city periphery and suburbs). Women who lived in the satellite city were also less likely to walk (OR: 0.53) compared to those who live in the central city, but the difference was not statistically significant. There was also no difference in walking among older women residing in the urban fringe with poor park areas access compared to those in the central city (OR: 0.98). Pairwise comparison between other clusters did not identify other significant differences.

In addition, the likelihood of women walking decreased with increasing age or increasing neighborhood block group share of live-alone population in poverty. Likelihood of walking increased for older women with more than 12 years of education compared to those with 12 years or less education (OR: 1.32) or who burned more than 2500 kcal per week through exercise compared to those who exercise less (OR: 2.49). On the other hand, the likelihood of walking as compared to the reference group decreased for women who rate their health fair, poor, or very poor (OR: 0.52); smoked 1–40 pack-years (OR: 0.88) or over 40 pack-years (OR: 0.66); were overweight (OR: 0.83), or obese (OR: 0.59); had a history of stroke (OR: 0.57).

## 4. Discussion

Few studies have either quantified built environment characteristics using high-resolution geographic unit or have used clusters to classify attributes of the built environment as urban forms. Our analysis pulls together various objective, high-resolution, and standardized measures to identify neighborhood clusters that portray the walking potential for individuals. Cluster analysis identified six homogeneous zones optimizing the joint distribution of built environment characteristics: accessibility to transit, land use mix, street connectivity, and residential density. The analytic process classified the Portland metro area into six unique urban-forms clusters: central city, city periphery, suburb, urban fringe with poor commercial area access, urban fringe with poor park area access, and satellite city. These urban-form clusters accounted for multiple built environment measures jointly without tying to an arbitrary administrative or neighborhood boundary which arguably fails to accurately represent the complex mix of urban forms.

Our analytic approach builds upon theory related to the urban built environment and physical activity; empirical use of built environment measures in combination to create a summary characterization of neighborhood type provided greater contextual meaning about the composition of different built environments without committing to a predefined neighborhood boundary. Our successful application of these methods to evaluate likelihood of walking in older women suggests potential utility for future studies. Although a variety of methods have been used to define and quantify characteristics of the built environment, challenges remain with the dissemination of spatial information at refined geographic units. We created objective and localized built environment measures using standardized geographic units that were not bigger than a city block. This measurement scale allowed for geographically relevant built environment measures that were linkable to individual-level data.

In previously published research, Riva and colleagues [10] used cluster analysis to identify exposure to built environment characteristics that promote physical activity such as access, land use, and density. Similar to our analysis, Riva et al. observed that the number of 10-minute episodes of walking was significantly higher in the central urban zone compared to the low density suburban zone. While the specific methods to quantify built environment variables varied slightly from our approach, they obtained similar findings suggesting that the methodology of cluster analysis is robust and useful. Differences in data availability and the specific ways in which variables can be operationalized will exist, but cluster analysis as a method appears to be able to identify meaningful areas with distinct characteristics.

In our analysis, older women living in neighborhoods characterized by high population density, high street connectivity, convenient access to amenities, especially transit and commercial areas were most likely to walk for exercise and transport. Our findings are consistent with previous studies that indicate that good infrastructure and design, such as availability of transit services and high street connectivity, encourage people to navigate around the area [20, 28, 34]. The existence of commercial businesses and parks appears to provide destinations that attract people to visit [2, 11, 26], and in turn, the high population density provides the capacity to support businesses and transportation services [2, 11, 20, 26]. It is reasonable to hypothesize that these components act similarly to support a greater likelihood of walking among older women. Comparison between the clusters suggested that access to destinations, such as park or commercial areas, increases the likelihood of walking. Residents in the urban fringe with poor access to commercial areas were more likely to walk compared to residents in the urban fringe with poor park access, suggesting that proximity to a commercial area might play a stronger role in promoting walking than proximity to a park in this age group.

Several limitations in the current study must be recognized. Given the cross-sectional nature of our study, it is not possible to determine whether association reflects a preference among active older women to live in the central city areas for reasons such as greater transportation options or better access to amenities. However, neighborhood selection is only one of the many factors affecting mobility of the older population. Generally, factors determining the mobility of the elderly population include life cycle events, environmental factors, and economic status [35]. Moreover, diversity in the older population, including age, gender, and resources, also accounts for varying housing preferences and mobility levels [3, 35–37]. Some literature suggests that an individual's preference may not be consistent with the actual living environment or affect walking habits [38, 39]. In other studies, intraurban migration pattern among elderly suggests that older adults who are wealthy and socially active tend to move away from central city, while more diverse individuals of lower income relocate within similar neighborhoods [40]. Also, neighborhood preferences may not play a strong role in the relocation decision among those who moved [3]. While self-selection cannot be ruled out, it may have limited impact on the observed association in our analysis. 

The age of our data may limit its generalizability to the present day. The geographic and health data used for this study were collected or recorded 26 years ago. The urban process and development experienced in Portland might have since altered the clusters and the composition of neighborhood factors. Some of the findings from the current study may not be directly applicable in contemporary sense due to changes in urban forms occurred since 1986; however, the findings and relative associations may be useful to areas that remained unchanged or have transformed to different urban forms identified. Additionally, the process is still relevant and useful for analysis of the association between built environment and walking using current data or data from other places and regions. This information provides general guidance and direction to changes in built environment that can encourage walking among older women.

## 5. Conclusions

In conclusion, we found that urban areas with the best access to transit services, close proximity to amenities such as businesses and parks, high street connectivity, and high population density were most likely to promote walking in older women. The use of cluster analysis synthesized the information to account for the complex interactions between multiple built environment attributes. The built environment characteristics of the central city were associated with increased utilitarian and leisure walking among older women relative to the city periphery, the suburb, or the urban fringe with poor commercial area access. The long-term benefits of shifting urban form to be more pedestrian-friendly are critically important with the aging population; changes in built environment would promote higher levels of physical activity among older adults and likely among people of other age groups. This change, in turn, may lower the risk of diseases related to the lack of physical activity in the population. Our analysis approach contributes to the theory related to urban-form typology and promotion of physical activity via the use of methods that quantify built environment characteristics without committing to a predefined neighborhood boundary. 

## Figures and Tables

**Table 1:** Summary of built environment attributes by cluster, Portland metro area.

Urban-form cluster	Median (interquartile range) distance in meters to the nearest:				Median (interquartile range) density per km^2 ^	
	Bus stop	Light rail station	Commercial area	Park	Intersections	Population
*Cluster 1*: central city	161 (80–241)	2968 (1451–5465)	228 (80–341)	402 (241–569)	111 (92–138)	2591 (2062–3027)
*Cluster 2*: city periphery	241 (114–402)	3817 (2037–7701)	332 (161–580)	433 (241–688)	53 (38–69)	1473 (1125–1793)
*Cluster 3*: suburb	433 (228–809)	12876 (9927–15253)	628 (322–995)	410 (180–688)	24 (10–42)	777 (407–1199)
*Cluster 4*: urban fringe with poor commercial area access	1778 (1048–2680)	11273 (7816–15331)	2255 (1835–2747)	724 (402–1074)	5 (0–13)	62 (0–334)
*Cluster 5*: urban fringe with poor park area access	1821 (1018–3030)	8459 (5413–12436)	1049 (613–1527)	1884 (1479–2421)	7 (2–17)	166 (10–367)
*Cluster 6*: satellite city	6112 (4474–8697)	23356 (21622–26551)	515 (241–900)	764 (433–1249)	26 (8–50)	641 (280–1093)

**Table 2:** Summary of self-reported daily walking by older women living in Portland metropolitan area according to urban-form cluster.

Urban-form cluster	Number of blocks walked per day					
	Mean	Standard deviation	Median	Minimum	Maximum	*N*
Walking for utilitarian purposes						

*Cluster 1*: central city	6.39	5.68	5	1	48	694
*Cluster 2*: city periphery	5.76	5.65	4	1	60	573
*Cluster 3*: suburb	6.67	7.13	4.5	1	48	170
*Cluster 4*: urban fringe with poor commercial area access	5.65	5.35	4	1	24	20
*Cluster 5*: urban fringe with poor park area access	6.95	7.60	4	1	36	41
*Cluster 6*: satellite city	5.33	4.30	5	1	15	9

Walking for leisure purposes						

*Cluster 1*: central city	14.69	10.85	12	1	82	492
*Cluster 2*: city periphery	13.88	10.30	12	1	72	457
*Cluster 3*: suburb	14.28	10.52	12	1	48	151
*Cluster 4*: urban fringe with poor commercial area access	9.79	8.40	8	1	24	14
*Cluster 5*: urban fringe with poor park area access	11.66	8.55	10	1	36	29
*Cluster 6*: satellite city	15.00	10.20	13.5	5	28	6

Walking for leisure and utilitarian purposes combined, excluding those who do not walk regularly						

*Cluster 1*: central city	14.60	13.36	11	1	102	799
*Cluster 2*: city periphery	13.98	12.88	10	1	96	690
*Cluster 3*: suburb	15.59	13.45	12	1	66	211
*Cluster 4*: urban fringe with poor commercial area access	10.00	9.65	5	1	30	25
*Cluster 5*: urban fringe with poor park area access	13.84	11.12	12	1	44	45
*Cluster 6*: satellite city	13.80	11.83	9.5	2	36	10

Walking for leisure and utilitarian purposes combined, including those who do not walk regularly						

*Cluster 1*: central city	13.24	13.41	9	0	102	881
*Cluster 2*: city periphery	12.26	12.91	8	0	96	787
*Cluster 3*: suburb	13.43	13.60	9	0	66	245
*Cluster 4*: urban fringe with poor commercial area access	8.93	9.63	4.5	0	30	28
*Cluster 5*: urban fringe with poor park area access	12.22	11.37	10	0	44	51
*Cluster 6*: satellite city	10.62	11.90	6	0	36	13

**Table 3:** Estimated odds ratios and 95% confidence intervals for the association of variables with walking.

Variable	Adjusted OR	95% CI	*P*-value
Built environment			<0.01
*Cluster 1*: central city	Reference		
*Cluster 2*: city periphery	0.69	(0.55, 0.85)	
*Cluster 3*: suburb	0.69	(0.50, 0.95)	
*Cluster 4*: urban fringe w/poor commercial area access	0.40	(0.18, 0.88)	
*Cluster 5*: urban fringe w/poor park area access	0.98	(0.52, 1.84)	
*Cluster 6*: satellite city	0.53	(0.16, 1.71)	
Percent live alone population in poverty, 1989	0.28	(0.11, 0.70)	<0.01
Age	0.95	(0.93, 0.96)	<0.01
Education level			<0.01
12 years or less	Reference		
More than 12 years	1.32	(1.08, 1.62)	
Self-rated health			<0.01
Excellent/good	Reference		
Fair/poor/very poor	0.52	(0.41, 0.67)	
Smoking			0.04
0 pack-years	Reference		
1–40 pack-years	0.88	(0.70, 1.11)	
More than 40 pack-years	0.66	(0.48, 0.92)	
Exercise (kcal per week)			<0.01
2500 kcal or less	Reference		
More than 2500 kcal	2.49	(1.84, 3.37)	
BMI			<0.01
Underweight/normal (less than 25.0)	Reference		
Overweight (25.0–29.9)	0.83	(0.66, 1.03)	
Obese (30.0 or above)	0.59	(0.46, 0.77)	
Stroke			0.01
No history of stroke	Reference		
With history of stroke	0.57	(0.37, 0.88)	
